# Progress in digestive endoscopy: Flexible Spectral Imaging Colour Enhancement (FICE)-technical review

**Published:** 2015

**Authors:** L Negreanu, CM Preda, D Ionescu, D Ferechide

**Affiliations:** * Internal Medicine II-Gastroenterology, University Emergency Hospital; “Carol Davila” University of Medicine and Pharmacy, Bucharest, Romania; ** Gastroenterology Department, Fundeni Clinical Institute; “Carol Davila” University of Medicine and Pharmacy, Bucharest, Romania; *** Internal Medicine Department, Monza Hospital; “Carol Davila” University of Medicine and Pharmacy, Bucharest, Romania; **** Physiology Department; “Carol Davila” University of Medicine and Pharmacy, Bucharest, Romania

**Keywords:** FICE, chromoendoscopy, minimal esophagitis, adenoma detection, Barrett’s esophagus

## Abstract

**Background.** A substantial advance in digestive endoscopy that has been made during the last decade is represented by digital chromoendoscopy, which was developed as a quicker and sometimes better alternative to the gold standard of dye spraying. Fujifilm developed a virtual coloration technique called Flexible spectral Imaging Color Enhancement (FICE).

FICE provides a better detection of lesions of “minimal” esophagitis, of dysplasia in Barrett’s esophagus and of squamous cell esophageal cancer. The use of FICE resulted in an improvement in the visualization of the early gastric cancer, being less invasive, and time consuming than the classic dye methods. Current evidence does not support FICE for screening purposes in colon cancer but it definitely improves characterization of colonic lesions. Its use in inflammatory bowel disease is still controversial and in video capsule endoscopy is considered a substantial progress.

**Conclusions.** The use of FICE endoscopy in routine clinical practice can increase the diagnostic yield and can provide a better characterization of lesions. Future studies to validate its use, the good choice of channels, and the “perfect indications” and to provide common definitions and classifications are necessary.

## Introduction

The digital chromoendoscopy, “a virtual coloration”, was developed as a quicker and sometimes better alternative to the gold standard of dye spraying.

Generally, white light can be visibly shaded into 7 colors, each carrying a different wavelength. The depth of mucosal penetration depends on the wavelength. Violet, which has the shortest wavelength, of 400 nm, does not penetrate the mucosa as deep as red, which has a longer wavelength, of 700 nm. Blue, green, and yellow are colors that have wavelengths in-between violet and red. Thus, the depths of penetration of these colors are ranged from 0.15 mm to 0.30 mm [**1**].

Hemoglobin is the main substance responsible for visible light absorption in the mucosa, with a peak absorption in the blue part (415 nm). Therefore, the blood vessels absorb 415 nm light and produce a dark brown color, which is different from the brighter surrounding mucosa, which reflects the blue light. Malignant or inflammatory areas which are highly vascular have this pattern.

The Olympus narrow band imaging (NBI) system uses narrow band light to increase the contrast. This has been shown to enhance the views, and it provides comparable diagnostic accuracy to classic chromoendoscopy [**2**,**3**].

Computed virtual chromoendoscopy from Fujifilm is called Flexible spectral Imaging Color Enhancement (FICE) and it reconstructs the image and displays what the mucosa would look like if illuminated by using a certain wavelength. The FICE processor generates a very large number of wavelength permutations with increments of 5 nm and has 10 pre-programmed settings. The advantage of the post-processed system is that all the preferred wavelengths are adjustable.

This review presents and discusses the clinical evidences of FICE technique, its use in current practice, its advantages and benefices but also its weak points. 

**Esophagus**

**Minimal esophagitis**

The majority of patients with gastroesophageal reflux disease (GERD) have non-erosive disease (NERD). However, a careful analysis by an experienced endoscopist may reveal subtle modifications even in some of these patients. 

The use of FICE improved the visualization of subtle lesions in non-erosive disease. NERD is classified into grade M (minimal change: erythema without sharp demarcation, whitish turbidity, and/ or invisibility of vessels due to these findings) and grade N (normal) in the modified Los Angeles classification [**4**].

The “triangular lesion”, defined as a triangular indentation into the squamous mucosa extended from the villiform columnar at the Z-line, was used as the endoscopic diagnostic criterion in 21 patients with typical symptoms of heartburn and/ or acid regurgitation examined by using FICE. Compared with the standard white light endoscopy, FICE provided higher sensitivity, negative predictive value, and accuracy in diagnosing minimal esophagitis [**4**]. 

In twenty-six NERD patients versus 31 controls, the NERD group had a higher proportion of minimal change, compared with the control group (77% and 48%, respectively) (P= 0.033). Minimal changes, such as erythema and whitish turbidity, which were detected by using conventional endoscopic images, showed up as navy blue and pink-white, respectively, in color using FICE images in the present FICE mode. The detection rates of minimal change using FICE images were significantly greater than those using conventional endoscopic images (for all readers) [**4**,**5**]. 

In conjunction with FICE, the magnification system of the endoscope may help enhance the details of the vascular pattern of esophageal mucosa. The increased numbers of tortuous and dilated intrapapillary capillary loops (IPCLs) is suggestive of reflux disease.

The FICE technique appears to provide similar results to the NBI system [**3**] but further validation is necessary. 

**Fig. 1a:**
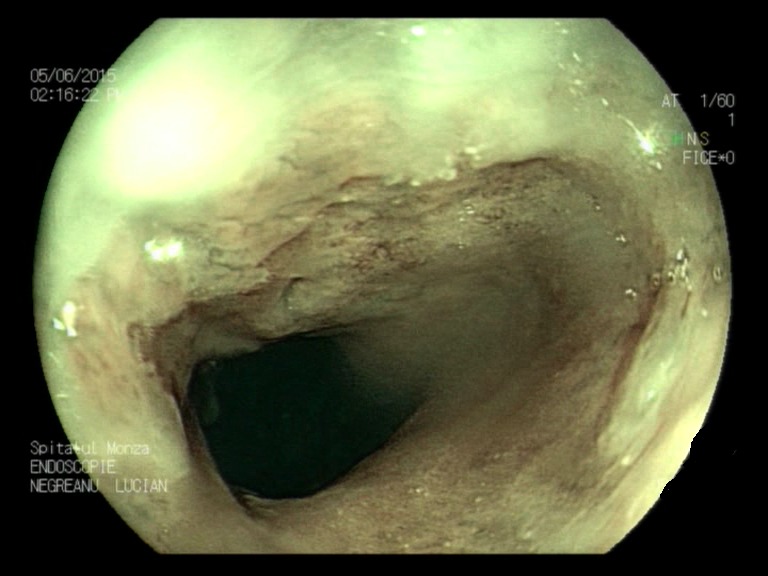
Minimal esophagitis – FICE filter 0 zoom

**Fig. 1b:**
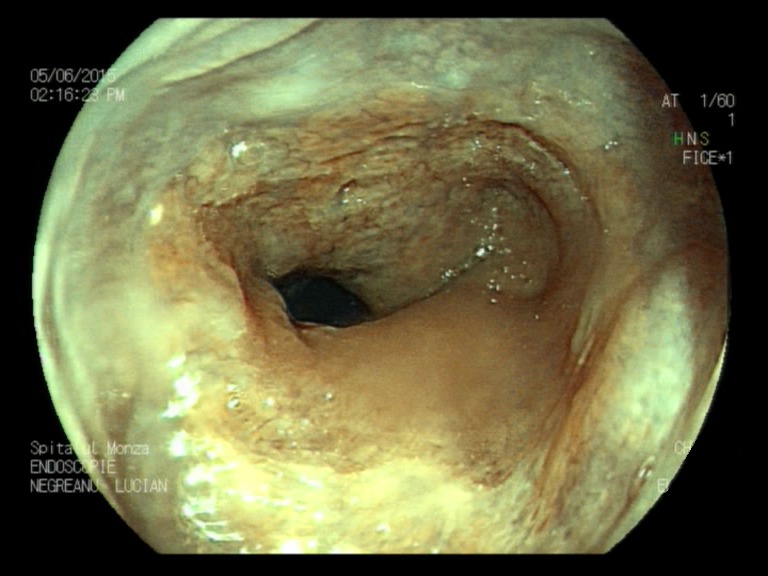
Minimal esophagitis – FICE filter 1

**Fig. 1c:**
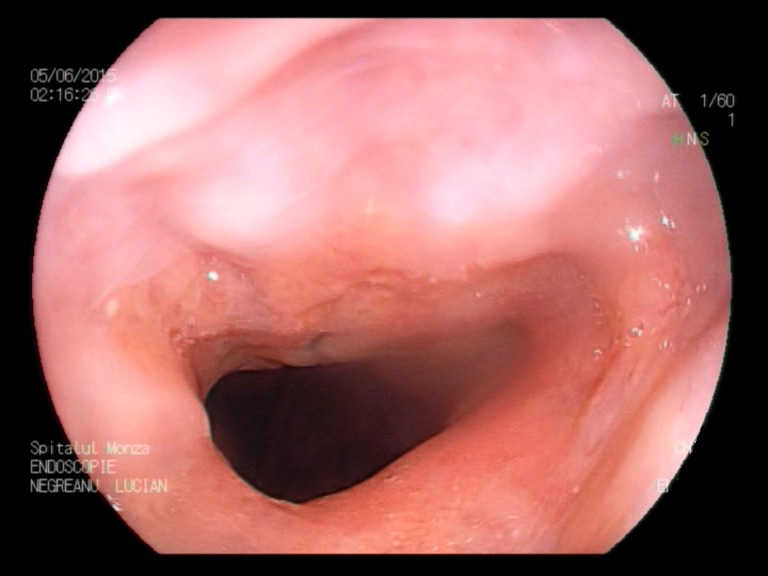
Minimal esophagitis – FICE white light

**Fig. 2a:**
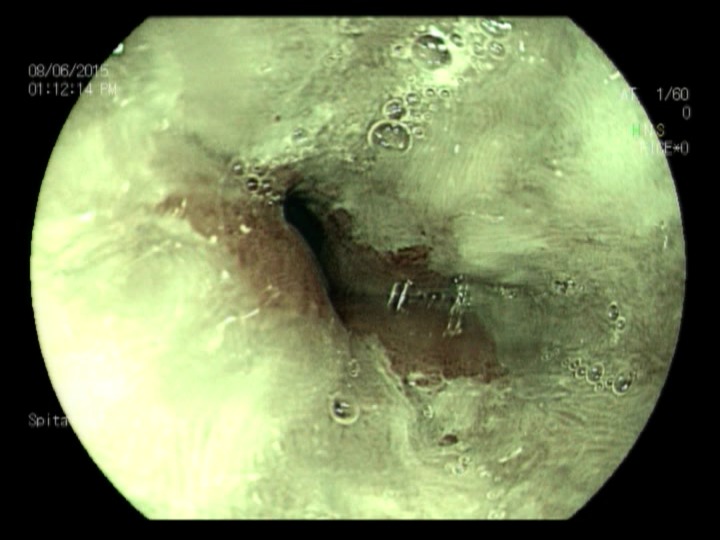
Minimal esophagitis – FICE filter 0

**Fig. 2b:**
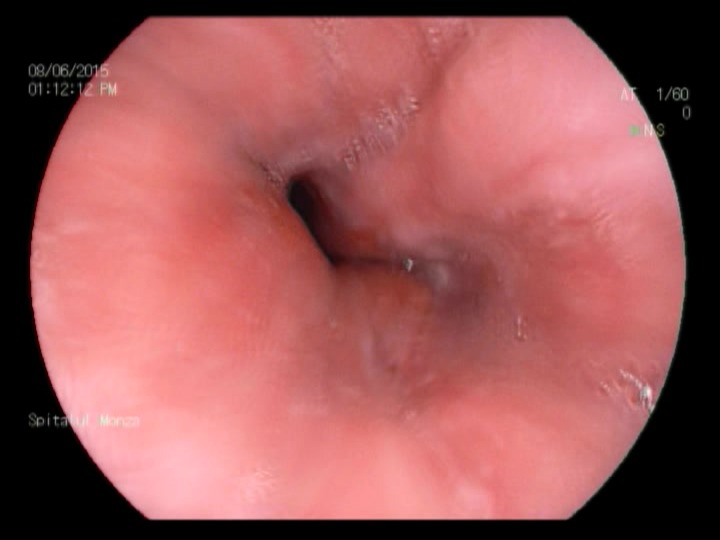
Minimal esophagitis – White light 2

**Fig. 3a:**
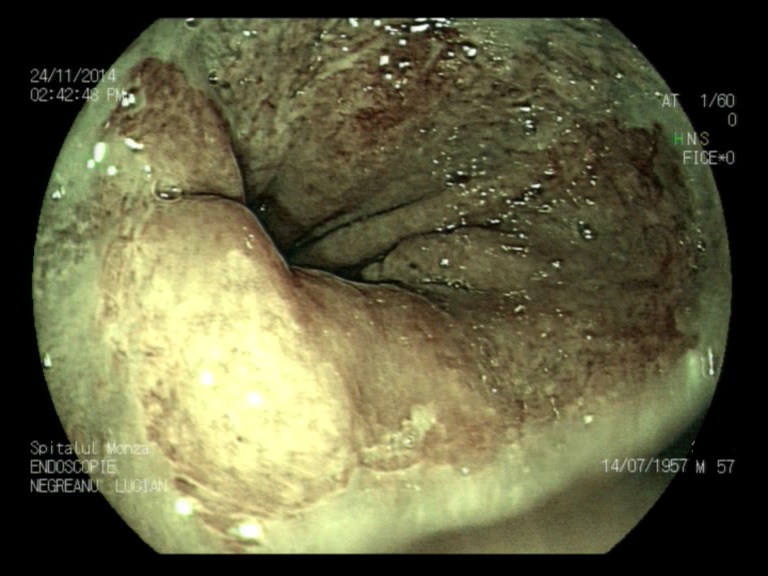
Minimal esophagitis – FICE

**Fig. 3b:**
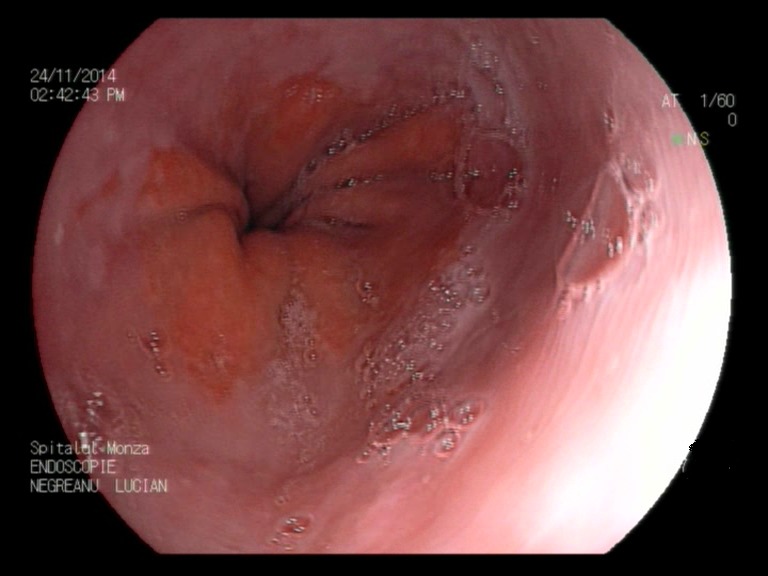
Minimal esophagitis – White light

**Barrett's esophagus**

Barrett's esophagus (BE) is usually monitored by using white-light endoscopy (WLE) and collecting random biopsy specimens according to the Prague recommendations. However, this approach does not definitively or consistently increases the detection of the areas of dysplasia; it is time consuming and is rarely applied strictly according to the recommendations in real life situations. 

A systematic review investigated whether the new imaging technologies can increase the diagnostic yield for the detection of neoplasia in patients with BE, compared with WLE and analysis of random biopsy specimens. Using Medline and Embase, an analysis of relevant peer-review studies included in the final analysis fourteen studies, with a total of 843 patients.

The advanced imaging techniques increased the diagnostic yield for the detection of dysplasia or cancer by 34% (95% CI, 20%-56%; P < .0001). A subgroup analysis showed that virtual chromoendoscopy significantly increased the diagnostic yield (RD, 0.34; 95% CI, 0.14-0.56; P < .0001) but with no significant difference between virtual chromoendoscopy and chromoendoscopy, based on Student t test analysis (P = .45) [**6**].

In a prospective pilot study, in a tertiary medical centre, FICE and acetic acid combined were compared with HD white light endoscopy in 18 patients with Barrett’s esophagus. High definition white light endoscopy showed an irregular mucosal pattern in 14% of the patients with high-grade dysplasia versus 100% when using acetic acid-FICE combined. Videos did not identify irregular vascular patterns using high definition white light endoscopy, while acetic acid-FICE combined visualized at least one abnormality in 86% of the cases [**7**].

**Squamous cell cancer**

In a Brazilian diagnostic test study that enrolled patients with head and neck squamous cell cancer, the performance of WLE and FICE for neoplasia detection was compared with the reference standard (lugol chromoscopy plus histology). The yields of WLE and FICE were similar for early squamous cell cancer detection [**8**]. In a recent Chinese study, in 257 patients with suspicious lesions of the esophagus, the lesions and the intrapapillary capillary loop were observed and compared with the pathologic diagnosis that was regarded as the golden standard. The positive rates of early esophageal squamous cell carcinoma were 92.6% and 88.9% as examined by FICE and Lugol chromoendoscopy (p>0.05), and 96.3% and 92.6% as examined by magnifying FICE and magnifying Lugol chromoendoscopy (p>0.05), respectively, but the magnifying FICE could observe the IPCL of the esophagus clearly. These findings led to the conclusion that Fujinon intelligent color enhancement and magnifying FICE are complements to Lugol chromoendoscopy and magnifying Lugol chromoendoscopy in the diagnosis of early esophageal lesions [**9**].

**Stomach**

In the last years, a lot of evidence has favored the use of FICE over white light endoscopy in detecting and characterizing gastric lesions. However, a lot of work is to be done in this area to improve the definitions and establish a common classification. 

A recent meta-analysis showed that the classification systems varied between studies and a single description of gastric mucosal features with HR-scopes or at least per technology will have to be agreed on. This systematic review on the current evidence on the diagnostic use of narrow band imaging (NBI), flexible spectral imaging color enhancement (FICE) and endoscopic image enhancement technology (i-scan) endoscopies for gastric precancerous and cancerous lesions included 38 studies [**10**]. 

Thirty-one studies were included for NBI and 7 studies for FICE assessment in this systematic review. No article was found meeting inclusion criteria for i-scan endoscopy. Gastric pattern descriptions have been proposed for NBI and FICE studies by gathering all descriptions in one single description. This is a start for the establishment of a common classification of lesions and a standardized endoscopic semiology with the new endoscopic techniques [**10**].

Only one study regarding the discrimination of non-neoplastic lesions, adenomas, and cancer of the stomach using magnifying endoscopy with flexible spectral imaging color enhancement was reported. A lesion suspected to be neoplastic using conventional endoscopy with magnification, was carefully described, and recorded according to the pit pattern (non-neoplastic lesions, adenoma, and cancer). The procedure was repeated three more times with FICE with channels 0, 2, and 4. In this study, FICE was proven to be useful in differentiating the type of lesions of the gastric mucosa with the degree of inter-observer agreement among the results of 0.42 for classic white light and 0.50-0.59 for FICE with each of the three different channels [**11**]. 

The usefulness of FICE with a specific wavelength in the assessment of early gastric cancers (EGC), and the relationship between the FICE visualization and blood vessels was first evaluated retrospectively in a series of 100 patients. The study concluded that FICE resulted in an improvement in the visualization of the EGC with the advantage of being less invasive and time consuming than the classic dye methods [**12**]. 

The efficacy of ultraslim endoscopy with flexible spectral imaging color enhancement (FICE) for the diagnosis of early gastric carcinoma was later confirmed in 20 consecutive patients in whom 22 gastric neoplasms were resected by endoscopic submucosal dissection (ESD). This study showed a clear benefit from FICE, which more clearly visualized tumor margin than did conventional endoscopy. The effect was more remarkable in discolored lesions (100.0 %, 3/ 3 lesions) [**13**]. 

These findings were confirmed by the work of another Japanese team using small-caliber endoscopy with the FICE system in the detection of depressed-type early gastric cancer. The study included eighty-two patients, diagnosed with depressed-type early gastric cancer by standard endoscopy and biopsy. The characteristic finding of the depressed-type early cancer in most cases was revealed as reddish lesions distinct from the surrounding yellowish non-cancerous area. No magnification was used. Compared with conventional white light images, the demarcation line between the cancerous lesion and the surrounding area was more easily recognized with FICE and the FICE system provided a better color contrast of depressed-type early gastric cancers than conventional small-caliber endoscopy [**14**,**15**]. 

An evaluation of the usefulness of flexible spectral imaging color enhancement with indigo carmine (I-FICE) in early gastric cancer (EGC) demarcation was realized in 29 patients with differentiated-type EGC. The demarcation margins were most easily recognized both subjectively and objectively using I-FICE images, followed by CE, FICE and WLE images [**16**]. 

**Automatic detection software-is it feasible?**

Accurate FICE-based diagnosis requires training and experience. In addition, there is a learning curve and objectivity is necessary. 

Thus, a software program that can identify gastric cancer quantitatively was developed. Further development of this system will allow a quantitative evaluation of mucosal gastric cancers on magnifying gastrointestinal endoscopy images obtained with FICE [**17**]. It will be interesting to see the development, validation and follow up of this software.

**Colonic Lesions and Colorectal Cancer Screening**

Colonoscopy is the gold standard method for colorectal cancer screening. Nonetheless, a substantial miss rate of lesions with conventional, white-light colonoscopy (WL) remains a challenge. By increasing the contrast of mucosa and mucosal lesions, FICE might lead to an improvement in colonic adenoma detection during colonoscopy, better characterization of lesions using the Kudo classification and the better correlation with the histologic results. However, current studies have yielded variable and conflicting results.

A large randomized trial was undertaken to determine whether FICE technology really enhances adenoma detection rate (ADR). In this prospective study performed in a multicenter private practice and hospital setting, involving 8 experienced examiners (>10,000 colonoscopies each), 1,318 patients were randomly assigned to colonoscopy with either FICE or white light imaging on instrument withdrawal. Of the colonoscopies, 68% were screening and 32% were diagnostic examinations. The primary outcome measure was the ADR [**18**]. There was no difference between the two groups in terms of general ADR (0.28 in both groups), the total number of adenomas (184 vs. 183), or the detection of subgroups of adenomas. The rate of identification of hyperplastic polyps was also the same in both groups (127 vs. 121; P=0.67). The results were the same for both the screening and the diagnostic colonoscopy subgroups. Withdrawal time was the same in both groups (8.4 vs. 8.3 min, P=0.55) [**19**].

Another single centre prospective randomized trial of tandem colonoscopy with either FICE (FICE-WL group) or WL (WL-FICE group) in 359 average-risk adults undergoing screening colonoscopy had as primary end point measure the difference in adenoma miss rates, and as the secondary outcome the adenoma detection rate [**19**]. The number of adenomas detected by FICE and WL was 123 and 107, respectively. The adenoma miss rate with FICE showed no significant difference compared to that of WL (6.6% vs. 8.3%, P = .59). Characteristics of lesions missed by the use of FICE were similar to those missed by the use of WL; 93% of overall missed polyps were < or =5 mm, and none were > or =1 cm. All missed adenomas were low grade and nonpedunculated. There was no significant difference between FICE and WL in adenoma detection rate (mean 0.64 vs. 0.55 per patient, P = .65) or in the percentage of patients with > or =1 adenoma (33.7% vs. 30.4%, P = .74). Although there is superiority for FICE, this did not reach statistical significance [**20**]. 

The ability of an endoscopist to predict small polyp histology during a screening colonoscopy using FICE was analyzed in a prospective study performed on 763 consecutive, asymptomatic subjects who were undergoing screening colonoscopy. Pit patterns and vascular patterns were used to predict the histology of 525 polyps (mean size, 4.5 ± 2.2 mm, 315 adenomas) of less than 10 mm. FICE with high magnification was better for differentiating the histology of small polyps during screening colonoscopy than FICE without high magnification, especially for diminutive polyps [**21**]. 

A comparison of FICE magnification to narrow-band imaging (NBI) magnification was realized by a Japanese team [**3**]. Flexible spectral imaging color enhancement or NBI magnification was performed to the visualize the surface and the vascular patterns of colorectal tumors, classified into 4 types: Type A, Type B, Type C1/ C2, and Type C3. A total of 235 colorectal tumors were examined. The correlations between classifications found by FICE or NBI magnification and histopathological diagnoses were examined. Image evaluation was validated by assessing inter-observer and intra-observer agreements on examinations. The classification of colorectal tumors by FICE magnification correlated well with the histopathological diagnoses, similar to the findings for NBI magnification [**3**]. 

ESGE suggests that virtual chromoendoscopy (NBI, FICE, i-SCAN) and conventional chromoendoscopy can be used, under strictly controlled conditions, for real-time optical diagnosis of diminutive (≤5mm) colorectal polyps to replace the histopathological diagnosis. The optical diagnosis has to be reported by using validated scales, must be adequately photodocumented, and can be performed only by experienced endoscopists who are adequately trained and audited (weak recommendation, high quality evidence) [**21**]. 

Based on these results, FICE cannot be routinely recommended for screening purposes but it can be useful for a better characterization of colonic polyps. 

**Inflammatory bowel disease**

In a recent article on expert centers on IBD in Romania, initially there was an agreement among experts that the access to virtual chromoendoscopy is mandatory in such a center [**22**]. However, an analysis of the literature did not show a superiority of the new methods over classical chromoendoscopy. Although the potential bene¬fits of the newer optical and digital dye-less chromoendoscopy techniques are substantial, only dye-based chromoendoscopy can currently be recommended to improve dysplasia detection in long-standing IBD [**23**,**24**].

In contrast, digital chromoendoscopy has the potential to quantify disease activity and mucosal healing in IBD [**23**,**24**]. 

**Video capsule endoscopy**

Recently, FICE has been increasingly used for the improvement of videocapsule endoscopy VCE image quality and the increase in the detection of lesions of the small bowel, especially angioectasias and erosions/ ulcerations.

The FICE settings 1 and 2 showed significantly superior detection ability over white light for vascular lesions. In the detection of erosive/ ulcerative lesions, FICE 2 was significantly superior to white light. For tumors, no significant improvement with FICE relative to classic white light images was observed [**25**].

In certain patients with OGIB, the use of FICE 1 might increase the detection of potentially bleeding lesions previously missed under conventional white light SBCE. A valuable strategy to obviate the need to repeat investigations in patients with OGIB, particularly for those who experience rebleeding should be the review of the first nondiagnostic SBCE using the FICE 1 filter. In a recent study, a significant increase of detection of angiomas and erosive lesions was done by using FICE 1(21% and 62% respectively) [**26**].

. In our daily routine, we use FICE 1 and blue mode routinely for evaluating lesions detected by using white light and also for a retrospective lecture of non diagnostic exams in obscure GI bleeding.

## Conclusions

The use of FICE in clinical practice of digestive endoscopy can increase the diagnostic yield and can provide a better characterization of lesions. Solid studies show that using FICE can lead to a substantial benefit in the detection of “minimal” esophagitis, a better detection of dysplasia in Barrett’s and oesophageal squamous cell cancer. FICE resulted in an improvement in the visualization of the early gastric cancer. Moreover, targeted biopsies can be conducted with an increased diagnostic yield. Although it can better predict polyp histology, it does not increase adenoma detection and current evidence does not support its routine use for screening purposes in colon cancer. The use of FICE seems promising in dysplasia detection and diagnostic of mucosal healing in ulcerative colitis and might be decisive for the diagnosis of vascular and erosive lesions of the small bowel during videocapsule endoscopy.

We need future studies to validate the good choice of channels, the “perfect FICE indications”, and the validation of common definitions and classifications.

**Acknowledgement**

Lucian Negreanu received an educational grant for young researchers from “Carol Davila” University of Medicine and Pharmacy and the bibliographical research was supported by this grant.

**Conflicts of interest**

The authors declare that they have no competing interests.
